# Self-reported preferences and barriers to continued professional development in primary care physicians: a cross-sectional web-based survey in Qatar

**DOI:** 10.1186/s12875-023-02235-x

**Published:** 2023-12-13

**Authors:** Deema Al-Sheikhly, Saima Ali, Phyllis Sui Muffuh Navti, Ziyad Riyad Mahfoud, Laudy Mattar, Samar Aboulsoud, Mohannad Khandakji, Lara Al Hakim, Thurayya Arayssi

**Affiliations:** 1 Medical Education and Continuing Professional Development Weill-Cornell Medicine- Qatar Education City, Doha, Qatar; 2 Division of Continuing professional development,, Weill-Cornell Medicine- Qatar, Doha, Qatar; 3 Population Health Sciences, Weill Cornell Medicine – Qatar, Doha, Qatar; 4 Internal Medicine, Cairo University, Cairo, Egypt; 5 Hamad Dental Center, Hamad Medical Corporation, Doha, Qatar; 6 Clinical Research Institute (CRI), American University of Beirut (AUB), Beirut, Lebanon; 7 Weill Cornell Medicine – Qatar, Doha, Qatar

**Keywords:** CPD, Lifelong learning, Primary care physicians, Barriers, Needs assessment

## Abstract

**Introduction:**

High quality and effective primary healthcare is a national priority in Qatar. Continuing professional development (CPD) for physicians is a cornerstone of this objective, yet little is known about physicians’ preferences or barriers to CPD participation.

**Method:**

A needs assessment was conducted using a cross-sectional web-based survey of primary care physicians registered with the Department of Healthcare Practitioners (DHP) between March and June 2017.

**Results:**

Two-hundred-and-eighty-one complete surveys were submitted representing physicians in both public (*N* = 129) and private sectors (*N* = 152). Physicians completed medical degrees and postgraduate training across multiple countries, and most had been practicing in Qatar for 5 years or less. ‘Activities during working hours’, ‘cost’ and ‘work commitments’ were the most common barriers. There was little consensus regarding the optimal timing of CPD activities, although public sector physicians were more likely to indicate weekend activities as a barrier to participation (30% vs. 9%). Over 90% of participants preferred traditional lectures, workshops, case-based sessions, small group and online self-paced learning as formats for CPD delivery, however alternative modes of delivery were also deemed acceptable (> 80% agreement).

**Conclusion:**

Understanding primary care physicians’ barriers and preferences is an essential component of a larger necessitated needs assessment of CPD in primary care practitioners in Qatar. Further research is warranted to understand the underlying beliefs driving physicians’ choices and the apparent variation between those working in the public and private sectors. CPD developers should consider approaches to mitigate perceived barriers and understand preferences to maximize the quality of participation.

**Supplementary Information:**

The online version contains supplementary material available at 10.1186/s12875-023-02235-x.

## Introduction

Though a relatively small country in the Arabian Gulf, Qatar has experienced rapid cultural and economic growth following the discovery of new energy sources in the 1970’s and is now one of the wealthiest nations in the world as measured by GDP per capita [1]. It also has one of the fastest growing populations in the world, attributed largely to the arrival of economic migrants who now constitute 80% of its increasingly diverse and multicultural population [2]. This growth has in turn led to significant developments in Qatar’s education and healthcare systems.

In 2008, Qatar proposed its National Vision for 2030 (QNV2030) outlining a series of long-term goals for the country. Human development according to the QNV2030 would enable a comprehensive and modern healthcare infrastructure that would allow access to all residents and citizens [3]. The QNV2030 guides national strategies aiming to implement policies and achieve the goals set out by the national vision. The National Health Strategy (2011–2016) and its subsequent strategies (2018–2022, PHCC) have been key drivers for change and reform in Qatar’s healthcare system [4–6]. The development and strengthening of primary care as the first and continuous point of contact for patients in Qatar is one such reform. The emphasis on primary care represents a shift from the previous healthcare approach, in which resources were primarily focused on secondary and specialized care, to a more holistic community-based model focused on prevention and health promotion [5, 6].

Both public and private primary health care services are available in Qatar. The publicly managed Primary Health Care Corporation (PHCC) is the largest provider, with 31 health centers across the country. There are also numerous private primary healthcare services that can be accessed using private health insurance or self-funding. Furthermore, the private sector’s capacity is set to expand under the National Health Strategy 2011–2022, with plans for universal health insurance coverage [7].

Central to delivering a world class primary healthcare system is the need for highly skilled primary care physicians. Qatar, like many countries within the GCC, has relied heavily on an expatriate workforce [8, 9]. While this has increased the number of practicing physicians, it is not without challenge. Several studies have demonstrated difficulties that physicians face when they have trained in a country other than the one they practice in. Some of the challenges include cultural differences, language and communication issues, and unfamiliarity with the healthcare system [9–13]. Efforts to address some of these issues include updating knowledge and skills via continuing professional development (CPD) [9, 13]. CPD is considered an essential component of high quality and effective medical practice [14, 15]. As a concept, it encompasses not only the continuous acquisition of medical knowledge and skills, but also a comprehensive range of managerial, leadership, ethical, social, and personal skills [16, 17]. It is essentially a lifelong process and importantly, allows healthcare professionals to maintain their competence and safety in practice in light of medical advancement and evolving scopes of practice.

Considering its significance in safe and effective health care, CPD has become increasingly mandatory for healthcare professionals and is frequently linked to revalidation and relicensing procedures for physicians [18–20]. Driven by the National Health Strategy 2011–2022, the Accreditation Section of the Department of Healthcare Professions (formerly the Qatar Council for Healthcare Practitioners (QCHP) and herein referred to as DHP) launched a national CME/CPD framework in 2016 [20]. The DHP is responsible for the regulation of all full and part-time healthcare practitioners in the state of Qatar in both the public and private sector. Following the introduction of the framework, all licensed healthcare practitioners (Physicians, Nurses, Pharmacists, Dentists, Allied Health Professionals, and Complementary Medicine Practitioners) currently working in Qatar are required to participate in accredited CPD activities to renew their practice license [20].

Despite an awareness of the need for lifelong learning, international research has demonstrated that physicians face multiple barriers to participating fully and effectively in CPD [21–28]. Furthermore, enforced CPD may also be perceived as an additional burden for physicians who may already feel overstretched [20–21, 27].

Since the introduction of the national CPD framework, there has been a paucity of research examining primary care physicians’ views regarding CPD in Qatar. Considering the relatively recent introduction of the national CPD framework, a rapidly changing healthcare system, and the multinational population and workforce, it is likely that primary care physicians may encounter unique challenges. It is therefore necessary to understand the potential barriers and preferences for CPD activities to maximize participation and ultimately achieve improved performance in primary care physicians and enhance patient outcomes.

As part of the service developmental and evaluation process, the DHP in collaboration with Weill Cornell Medicine-Qatar and the Royal College of Physicians and Surgeons of Canada conducted a CPD needs assessment of primary care physicians registered in the state of Qatar. The present paper reports the findings related to the barriers and preferences for CPD identified via this needs assessment.

## Method

### Study design and participants

As part of a service development initiative, a cross-sectional online survey was conducted between March and June 2017. An email invitation to complete the survey was sent via the DHP in Qatar to all primary care physicians registered on their database. The invitation included the purpose of data collection, a statement regarding voluntary participation, and a link to the anonymous online survey hosted on the Alchemer (formally SurveyGizmo) platform (www.alchemer.com).

Physicians working in either public or private sectors were eligible to participate if they were currently registered with the DHP and contactable via the email associated with their registration during the timeframe of data collection. Participant data were excluded from the present analysis if information regarding their work setting (public or private) was not available.

The project was granted ethical exemption following review by the Institutional Review Board at Weill Cornell Medicine-Qatar.

### Online survey

The online survey for the needs assessment was developed by the DHP and the Royal College of Physicians and Surgeons of Canada (RCPSC) in collaboration with Weill Cornell Medicine-Qatar, following a review of the literature and professional opinion. The survey comprised three sections: (1) personal and demographic details, (2) administrative details and (3) clinical details. The present study includes data from the personal and demographic and administrative sections (Supplementary material 1).

Personal and demographic details included: Age group (25–35, 36–45, 46–55, 56–65 and 66+); Gender (Male or Female); Country in which the Medical Degree (MD) was completed; Country or Countries in which postgraduate training was completed; Specialty training, Board certification; Total years in practice (1–5, 6–10, 11–20, 20+); Years of practice in Qatar (1–5, 6–10, 11–20, 20+); and Specialty (family physician, internal medicine specialist, general practitioner, general practitioner with special interest, or ‘other’).

Administrative details included: Category to best describe clinical work (private clinic, private hospital, Primary Healthcare Center, university/academic facility, or ‘other’); Preference for CPD announcement method (Email/ Newsletter, Facebook, Newspaper advertisement, Phone Call, SMS (Text message), Twitter, via employer, or the DHP website with participants asked to rank these methods from 1 to 10; Barriers to CPD attendance (participants to select all that apply from the following list: ‘activities are held during working hours’, ‘activities are held during weekends’, ‘cost’, ‘current activities are of no interest to me’, ‘lack of accessible venue’, ‘lack of administrative support/ resources’, ‘lack of time’, ‘work commitments’, ‘other, please specify’); Preferred time to attend CPD activities (weekday half-day, weekday full-day, weekend half-day, weekend full-day); and Preferred CPD format (‘case-based presentations’, ‘hands on lab’, ‘lectures’, ‘panel discussions’, ‘questions and answers’, ‘workshops’, ‘face to face’, ‘live online presentations and discussion on your computer’, ‘online self-learning module’, and ‘blended’) with participants asked to rate each format on a 5-point Likert scale ranging from 1 strongly disagree to 5 strongly agree).

### Data analysis

Data were analyzed using IBM SPSS software (version 20, Armonk NY, USA). Frequency distributions (that is the number and percentage) were used to summarize each categorical variable for the whole sample and separately for physicians working in the public and private settings. Chi-square tests (or Fisher’s exact tests when expected cell counts fell below five) were used to test differences in demographic and work-related variables as well as in each barrier to CPD activity, the preferred timing of activity, and the preferences for CPD format between physicians working in the public verses private sector.

Country data was categorized by region or continent. The World Bank definition was adopted to create a category for the Middle East and North Africa (MENA) region [28].

As data for the preferred method of CPD announcement were ranked, the total number of participants selecting 1 (highest ranking) for each method were calculated and presented with a percentage of the total number.

Answers for the preferred format of CPD activities were dichotomized to a yes/no format by collapsing responses 1–2 (strongly disagree and disagree) and 3–5 (neutral, agree, and strongly agree) from the original 5-point scale. The rationale for dichotomization between 2 and 3 being that those who answered 3 or above had not opposed this format of delivery and findings did not differ significantly compared to the 5 point scale”.

## Results

Over twelve weeks, 350 surveys were submitted online. Of these, 69 were excluded as the work setting could not be ascertained, leaving a final sample of 281. A response rate of 21.5% was estimated based on 1,304 registered primary care physicians in the public and private sector in Qatar in 2017 [6].

Table 1 shows respondents’ demographic details, as well as their diverse educational and practice backgrounds. 62% of respondents were male, and the largest proportion (38%) were between 36 and 45 years old. Participants had completed MDs in one of 40 different countries of which most were in Asia (44.5%) and the MENA region (40.9%). Within the Asian countries (data not presented in table), the largest proportion of physicians completed their MDs in India (*N* = 60, 21.4%), the Philippines (*N* = 34, 12.1%) and Pakistan (*N* = 27, 9.6%). From those in the MENA region, most completed their MDs in Egypt (*N* = 61, 27.1%), Jordan (*N* = 10, 3.6%), Tunisia (*N* = 10, 3.6%), Syria (*N* = 10, 3.2%), and Sudan (*N* = 10, 3.2%).

**Table 1:** Demographic, educational and practice characteristics of primary care physicians in Qatar

Variable	Total *N* (% of total)	*N* (% within each setting)		*P*-value
	*N* = 281	Private *N* = 152	Public *N* = 129	
**Age**				
25–35	62 (22.1)	42 (27.6)	20 (15.5)	0.074
36–45	107 (38.1)	55 (36.2)	52 (40.3)	
46–55	75 (26.7)	33 (21.7)	42 (32.6)	
56–65+	37 (13.2)	22 (14.5)	15 (11.7)	
**Gender**				
Male	173 (61.6)	87 (57.2)	86 (66.7)	0.105
Female	108 (38.4)	65 (42.8)	43 (33.3)	
**MD region**				
MENA	115 (40.9)	55 (36.2)	60 (46.5)	0.040*
Asia	125 (44.5)	79 (52.0)	46 (35.7)	
Europe	34 (12.1)	14 (9.2)	20 (15.5)	
Other	7 (2.5)	4 (2.6)	3 (2.3)	
**Postgraduate training**				
N	294	159	129	
MENA	117 (42.2)	59 (37.1)	65 (48.2)	< 0.001*
Asia	100 (34.0)	73 (45.9)	27 (20.0)	
Europe	61 (20.8)	24 (15.1)	37 (27.4)	
Other	9 (3.0)	3 (1.9)	6 (4.4)	
**Years of practice**				
1–5	17 (6.0)	10 (6.6)	7 (5.4)	0.936
6–10	68 (24.2)	35 (23.0)	33 (25.6)	
11–20	101(35.9%)	56 (36.8)	45 (34.9)	
Over 20	95 (33.8%)	51 (33.1)	44 (34.1)	
**Years in Qatar**				
1–5	164 (58.4)	90 (59.2)	74 (57.4)	0.610
6–10	64 (22.8)	32 (21.1)	32 (24.8)	
11–20+	30 (10.7)	15 (9.9)	15 (11.6)	
Over 20	23 (8.2)	15 (9.9)	8 (6.2)	
**Specialty training**				
Yes	174 (62.6)	85 (56.7)	89 (69.5)	
**Specialty**				
Family Physician	48 (17.1)	7 (4.6)	41 (31.8)	< 0.001*
General Practitioner	67 (50.2)	91 (60.3)	50 (38.8)	
General Practitioner (SI) ^**†**^	79 (28.1)	51 (33.6)	28 (21.7)	
Internal Medicine	13 (4.6)	3 (2.0)	10 (7.8)	
**Work Setting**				
Private Clinic	93 (33.1)	93 (61.2)	--	< 0.001*
Private Hospital	59 (21.0)	59 (38.8)	**--**	
Primary Healthcare Center	108 (38.4)	**--**	108 (83.7)	
Academic Facility	21 (7.5)	**--**	21 (16.3)	

*p-value < 0.05 significant differences between the two groups. †General Practitioner with special interest

Similarly, postgraduate training was also completed across a diverse list of 37 countries. Eighteen respondents completed postgraduate training in more than one country. Over half (58%) of participants completed their postgraduate training outside of the MENA region. In terms of individual countries (data not presented in table), the largest proportion received training in Egypt (*N* = 51, 17%) followed by India (*N* = 39, 13.2%), the UK (*N* = 33, 11.2%), and Qatar (*N* = 28, 9.5%).

Although the largest proportion of participants had been in practice for 11–20 years, most physicians (58%) had only been practicing in Qatar for around 1–5 years.

### Public and private sector physicians

One hundred and fifty-two primary care physicians were working in the private sector and 129 in public sector settings. There were no age or gender differences between those working in public and private settings. However, significant differences were observed between the two groups in both the country where the MD was completed (*P* = 0.040) as well as further training (*P* < 0.001). A higher proportion of physicians working in the public sector completed their MDs in the MENA region (46.5% vs. 36.2%) and in Europe (15.5% vs. 9.2%). A larger proportion of private sector physicians completed their MDs in Asia (52% vs. 35.7%). There were also significant differences in specialty training, which was higher in the public sector (*p* = 0.027), and in type of specialty (*p* < 0.001), with significantly more family medicine physicians working in public settings. There were no significant differences in the number of years in practice and the number of years in Qatar between the two groups.

### Barriers to taking part in CPD

The most reported barriers to CPD activities were ‘activities held during working hours’ (65.1%), followed by the ‘cost’ of activities (44.1%), and ‘work commitments’ (38.1%) (Table 2). Compared to public physicians, significantly more physicians working in the private sector, reported “cost” as a barrier (50.5% vs. 36.4%). On the other hand, significantly more physicians working in the public sector reported ‘activities being held during the weekends’ (29.5% vs. 9.9%) and ‘lack of time’ (38.8% vs. 20.4%) as being barriers to taking part in CPD.

**Table 2:** Number and percentage of participants identifying barrier to CPD by work setting

Barrier	Total N (%) *N* = 281	Work Setting N (%)		*P* value
		Private *N* = 152	Public *N* = 129	
Activities are held during working hours	183 (65.1)	99 (65.1)	84 (65.1)	0.998
Activities are held during the weekends	53 (18.9)	15 (9.9)	38 (29.5)	< 0.001*
Cost	124 (44.1)	77 (50.7)	47 (36.4)	0.017*
Current activities are of no interest to me	41 (14.6)	26 (17.1)	15 (11.6)	0.195
Lack of accessible venue	36 (12.8)	12 (15.8)	24 (9.3)	0.105
Lack of administrative support	57 (20.3)	33 (21.7)	24 (18.6)	0.519
Lack of time	81 (28.8)	31 (20.4)	50 (38.8)	< 0.001*
Work commitments	107 (38.1)	53 (34.9)	54 (41.9)	0.229

*p-value < 0.05 significant differences between the two groups.

### Preferences (announcement, timing, and format)

‘Via email’ was the highest ranked method of preferred CPD announcement with approximately half of participants choosing this method (Table 3). The next most popular method of communication was ‘text message’ (10%), followed by Twitter (7.8%), the DHP website (7.1%), and Facebook (6%). The preferred methods of announcement were not significantly different between physicians in the public and private sector except for ‘phone calls,’ which was significantly higher among the physicians in the private sector (Table 3).

**Table 3:** Number and percentage of participants selecting the highest ranking value per CPD announcement method

Announcement Method	Total *N* = 281	Private *N* = 152	Public *N* = 129	*p*-value
Email	148 (52.7)	77 (51.0)	71 (55.0)	0.464
Facebook	17 (6.1)	10 (6.6)	7 (5.4)	0.676
Newspaper	9 (3.2)	6 (4.0)	3 (2.3)	0.436
Phone call	16 (5.7)	14 (9.3)	2 (1.6)	0.006*
Text message	28 (10.0)	17 (11.3)	11 (8.5)	0.448
Twitter	22 (7.9)	10 (6.6)	12 (9.3)	0.406
Employer	11 (3.9)	6 (4.0)	5 (3.9)	0.967
DHP‡	20 (7.1)	8 (5.3)	12 (9.3)	0.195

*p-value < 0.05 significant differences between the two groups. †Department of Healthcare Professions.

Almost 45% of participants preferred that CPD be held for a half-day during the weekends, with no significant difference reported between public vs. private sector (Table 4). However, almost 30% of physicians working in the private sector preferred CPD activities to be held for a full day on the weekend, significantly greater than the 17% reported among physicians in the public sector.

**Table 4:** Preferred time to attend activities: number and percentage of participants per work setting (public vs. private)

Preferred time to attend CPD activities	Total	Setting N (%)		*P*
	*N* = 281	Private *N* = 151	Public *N* = 129	
Weekdays-Full day	41 (14.6)	17 (11.2)	24 (18.6)	0.079
Weekdays-Half day	60 (21.4)	33 (21.7)	27 (20.9)	0.874
Weekend-Full Day	66 (23.5)	44 (28.9)	22 (17.1)	0.019*
Weekend-Half day	126 (44.8)	68 (44.7)	58 (45.0)	0.970

*p-value < 0.05 significant differences between the two groups.

Although all formats for CPD delivery were considered acceptable (> 80% agreement), the most preferred were lectures and workshops (94.3% each), followed by case-based learning (92.9%), small group learning sessions (92.2%), and online self-learning modules (91.1%). No significant differences were observed between physicians working in the public and private sectors (Table 5).

**Table 5:** Preferred format for CME/CPD activities. Number and percentage of physicians’ agreement

CPD activities format	Total (%)	Private (%)	Public (%)	P
Hands on Lab	245 (87.2)	134 (88.2)	111 (86.0)	0.598
Case based	261 (92.9)	142 (93.4)	119 (92.2)	0.703
Face to Face	248 (88.3)	134 (88.2)	114 (88.4)	0.956
Lectures	265 (94.3)	146 (96.1)	119 (92.2)	0.170
Live broadcast of presentations on site	238 (84.7)	134 (88.2)	104 (80.6)	0.080
Live online presentations and discussion on your computer	234 (83.3)	130 (85.5)	104 (80.6)	0.272
Online self-learning module	256 (91.1)	139 (91.4)	117 (90.7)	0.826
Panel discussions	240 (85.4)	132 (86.8)	108 (83.7)	0.460
Questions and answers	251 (89.3)	136 (89.5)	115 (89.1)	0.930
Small group learning sessions	259 (92.2)	139 (91.4)	120 (93.0)	0.624
Workshops	265 (94.3)	146 (96.1)	119 (92.2)	0.170
Blended	241 (85.8)	132 (86.8)	109 (84.5)	0.575

## Discussion

The diversity of countries in which participants trained and obtained their medical degrees, as well as the relatively short time practicing in Qatar, reflects the recent and rapid growth in the primary health care workforce via expatriate recruitment. While this has been invaluable for the development and growth of Qatar’s healthcare system, it also re-emphasizes the need for systematic and continuous support for physicians who bring knowledge from heterogeneous curriculums and differing experiences with CPD. The present study contributes to the literature as the first to identify the barriers and preferences for CPD in primary care physicians in Qatar.

Activities held during working hours, cost, and work commitments were the most cited barriers to CPD, and likely indicate an increased workload and pressure faced by primary care physicians in Qatar. These findings are consistent with a large body of international literature that has often cited factors relating to time and workload as common barriers to CPD engagement, including studies in Saudi Arabia, Jordan, Oman, and Bahrain [31–34] and beyond the Middle East region (30–22, 26–27). Findings regarding the financial barriers to CPD are less consistent across studies (21–22, 35) and may reflect differences in funding and reimbursement for CPD across countries, or the types of CPD that physicians engage in.

In the present study, the cost of CPD was also more often cited as a barrier by physicians working in the private sector compared to those in the public sector, whereas time constraints and activities being held at the weekends posed a particular challenge to physicians working in the public sector. Further research is warranted to understand these discrepancies by work settings and whether they reflect differences in demographic profile between physicians employed in each setting and/or differences in the provision of protected time and financial support for activities.

The parallels and discrepancies of our findings with global studies provides a comparative perspective which contribute to international discourse as well as a broader understanding of professional development needs in primary care. Future international research is also necessary to scrutinize disparities between public and private sector physicians in order to comprehensively understand the nuanced dynamics influencing professional development in diverse healthcare settings worldwide.

Research has demonstrated that physicians may struggle with finding relevant opportunities for CPD and have called for better outreach and publicity of courses [36]. In the present study, emails were the most preferred method to be informed about CPD activities for just over half of the participants. It is possible that the email invitation of this survey could bias towards physicians who are more receptive to email communication; however, physicians also showed preference to a variety of alternative methods, including text messages, social media, and direct telephone calls. A multimodal approach for CPD providers and marketing teams may therefore be more effective in terms of publicizing upcoming CPD events.

Our findings demonstrate that primary care physicians may also be amenable to a variety of CPD delivery modalities, although the most preferred methods largely included live face-to-face approaches, as well as self-paced online formats. These findings are in line with previous studies that have shown physicians prefer live learning due to the opportunity to take time away from practice and interact with colleagues, as well as a belief that subjects are taught more effectively using face to face methods [33, 36–42]. The perceived appeal of self-paced online learning, on the other hand, included greater flexibility in terms of scheduling, as well as lower cost (40–41). Finally, passive strategies, such as attendance at conferences and lectures, may also be considered convenient and cost-effective methods for staying current and preparing for accreditation [41].

Although physician preference remains an important factor for participation in activities, ultimately the goal of CPD is to improve outcomes for both physicians and the patients they serve. A plethora of literature has demonstrated the positive impact of CPD on physician knowledge, competence, performance, and patient health outcomes (15, 43–44). However, meta-analyses have also shown variance in the effectiveness of CPD in improving physician performance and patient outcomes according to the methods of implementation [15, 44] with larger effect sizes demonstrated when strategies are interactive such as case-based training, and small group learning [44]. Furthermore, although traditionally passive methods of CPD, such as lectures and didactic presentations, may not be as effective in terms of changes in physician behavior or patient outcomes, a positive association with physician knowledge and awareness has been demonstrated [44].

The findings from the present study in the context of this literature suggest that CPD providers in Qatar may have some flexibility in selecting from a range of methods for CPD that are both acceptable and appropriate for the course content and outcomes desired. Providers must, however, face the challenge of mitigating barriers to participation to reduce the perceived burden of activities and fully engage practitioners.

Meta-analyses examining effective components of CPD delivery formats have also demonstrated that increased course duration and continued contact with material over time provide a greater effect on outcomes and sustained change [15, 44]. These findings come at odds with barriers identified in the present study, namely the perceived lack of time and question of cost. Online learning formats may offer a promising solution, with the potential to save time and offer cost-effectiveness via eliminating costs for travel and overnight stays (40–41, 45). The delivery of these methods has become increasingly sophisticated and offers the potential to include adult learning approaches and multifaceted learning methods, allowing opportunities for practical problem solving and interactive discussion [42, 45]. Importantly, online formats have demonstrated similar outcomes to live face-to-face methods in terms of knowledge, engagement with content, and patient health outcomes [42, 45].

As with many countries that faced the challenges of social distancing, the COVID-19 pandemic catapulted the use of online delivery methods for CPD in Qatar. Local CPD providers were able to deliver accredited training as well as up to date information regarding COVID-19 to a record number of attendees [46, 47]. Common disadvantages cited by physicians in the literature include a lack of active participation and interaction between learners and facilitators. Moreover, technology-related and logistical issues such as camera placement, poor delivery format, and lack of coordination have also contributed to a less favourable experience with online learning [45]. However, there is currently a lack of literature from Qatar, so further research is needed to evaluate the use of various online formats in terms of provider and participant experience and the effectiveness on user and patient outcomes.

Several limitations to the present study should be acknowledged. Firstly, the modality of administrating the self-report survey in addition to the short response period may have biased towards early responders, in addition to physicians who are more comfortable with participating in an online format.

Although the study sample size is reasonable for a descriptive study, it might not have provided enough power to detect differences between setting that are sizeable. Future research endeavours may thus benefit from a comprehensive sample size analysis to ensure adequate power for detecting differences between physicians employed within each setting.

It is also possible that physicians may encounter barriers or have preference for CPD formats that were not captured within the present survey. Although an open-ended response option was provided, there was minimum engagement with this item, and as such, no additional factors or issues were identified. Furthermore, our analysis did not account for the potentially moderating impact of age, gender, or culture on physicians’ choices or barriers, particularly for specific delivery formats. Further qualitative research could provide more in depth and valuable insight into the factors which influence physicians’ choices, and how these factors may relate to motivation, engagement, and emotional experience with CPD, all of which may influence knowledge and skills acquisition [48].

This study was conducted prior to the COVID-19 pandemic. It is possible that physicians’ experiences and exposure to online content may now call their previous beliefs, preferences, and barriers to CPD into reconsideration. Since the COVID-19 era, the proliferation of online teaching methods and virtual encounters has also been evident at the undergraduate level. It would therefore be worthwhile to evaluate barriers and preferences for CPD modalities periodically in order to capture potential shifts in preferences post-COVID-19 as well as generationally. Data from the present study will allow useful comparison.

Despite these limitations, the present study represents the first report of the barriers and preferences to CPD in a national sample of physicians in both the public and private sectors in Qatar and is an important first step to towards a necessary national needs assessment for CPD in primary care in Qatar.

## Conclusion

Primary care physicians show preference towards a range of learning modalities and formats for CPD, however, ‘lack of time’ and ‘cost’ remain salient barriers to participation. It is necessary to invest in high-quality research to understand the underlying beliefs and motivations behind the CPD choices in this culturally diverse group of physicians, and to determine how different modalities and formats for CPD could be leveraged to potentially minimise barriers to participation and provide equitable access to all.

### Electronic supplementary material

Below is the link to the electronic supplementary material.

Supplementary Material 1

## Data Availability

The datasets used and/or analysed during the current study are available from the corresponding author on reasonable request.
