# Supplementary material for: Self-reported preferences and barriers to continued professional development in primary care physicians: a cross-sectional web-based survey in Qatar
Source: BMC Prim Care. 2023 Dec 13;24:273. doi: 10.1186/s12875-023-02235-x (PMC10717340; doi:10.1186/s12875-023-02235-x)
Supplement: Supplementary file 1 — Supplementary Material 1 [file 12875_2023_2235_MOESM1_ESM.docx]

### **Personal Details**

1. **Please indicate your age**

25-35

36-45

46-55

56-65

66 above

1. **Gender**

Male

Female

### **In which Country did you complete your medical degree**

### Drop down menu

### **In which Countries did you pursue post graduate training (click all that applies)**

### Drop down menu

### **Have you completed a specialty-training program? Yes/No**

### Yes

### No

### **If Yes, Please indicate which Board Certification/specialty certification you hold.**

### American Board of Medical Specialties

### Arab Board of Health Specializations

### Medical Council of Canada

### Medical Council of India

### Oman Medical Specialty Board

### Royal College of Physician’s and Surgeons of Canada

### Royal Colleges of Physicians of the UK

### Saudi Commission for Health Specialties

### Not Board Certified

### Other – Please list

1. **What is the total number of years you have been in practice**

1-5

6-10

11-20

Over 20

1. **How many years have you been in practice in Qatar**

1-5

6-10

11-20

Over 20

### **Please list your specialty (**Drop down menu)

Family Physician

Internal Medicine Specialist

General Practitioner (GP)

GP Special Interest in Anesthesiology

GP Special Interest in Cardiology

GP Special Interest in Dermatologiy

GP Special Interest in ENT

GP Special Interest in Internal Medicine

GP Special Interest in Neurology

GP Special Interest in Obstetrics/ Gynecology

GP Special Interest in Ophthalmology

GP Special Interest in Orthopedic Surgery

GP Special Interest in Pediatrics

GP Special Interest in Psychiatry

GP Special Interest in Radiology

GP Special Interest in Surgery

GP Special Interest in Urology

Other

###

### **Administrative Details**

1. **Which category best describes where you do most of your clinical work?**

Private Clinic

Private Hospital

Primary Healthcare Center

University/Academic Facility

Other (please specify)

1. **What is the best way for us to announce future Continuing Professional Development (CPD) activities (By order of preference)?**

Email / Newsletter

Facebook

Newspaper Advertisement

Phone Call

SMS

Twitter

Via your Employer

Weill Cornell Medicine-Qatar CPD Website

QCHP ePortfolio

1. **What are the main barriers preventing you from attending CPD activities? (Click all that apply)**

Activities are held during working hours

Activities are held over weekends

Cost

Current activities are of no interest to me

Lack of accessible venue

Lack of administrative support/resources

Lack of time

Work commitments

Other (please specify)

1. **What is your preferred time to attend activities? (Click all that apply)**

Weekdays-Full day

Weekdays-Half day

Weekends-Full day

Weekends-Half day

Other (please specify)

1. **I prefer CME/CPD activities to be delivered in this format**

Case-based Presentations

Hands on Lab

Lectures

Panel Discussions

Questions and Answers

Workshops

Face to face

Live online presentations and discussion on your computer

Online self-learning module

Blended
